# Supplementary material for: Development and validation of the Self-Harm Screening Inventory (SHSI) for adolescents
Source: PLoS One. 2022 Feb 10;17(2):e0262723. doi: 10.1371/journal.pone.0262723 (PMC8830763; doi:10.1371/journal.pone.0262723)
Supplement: Attachment — Submitted filename: Response to the Reviewers-final.docx [file pone.0262723.s003.docx]

**Response to the Reviewers**

Dear Prof. Paola Gremigni,

I wish to resubmit an original article for publication in *PLOS ONE*, titled “**Development and validation of the Self-Harm Screening Inventory (SHSI) for Adolescents.”** The manuscript ID is PONE-D-21-17797.

The manuscript has been rechecked and the appropriate changes have been made in accordance with the reviewers’ suggestions. The responses to their comments have also been drafted and attached.

I thank the editor and the reviewers for their thoughtful suggestions and insights, which have enriched the manuscript, and helped in producing a better and more balanced account of the study. I hope that the revised manuscript is now suitable for publication in your journal.

I have also attached the certificate of English editing below.

I thank you for your consideration, and am looking forward to hearing from you.

Sincerely,

Jong-Sun Lee

Department of Psychology, Kangwon National University

Chuncheon-si 24341, Korea

jongsunlee@kangwon.ac.kr

Journal Requirements:

When submitting your revision, we need you to address these additional requirements.

1. Please ensure that your manuscript meets PLOS ONE's style requirements, including those for file naming. The PLOS ONE style templates can be found at

<https://journals.plos.org/plosone/s/file?id=wjVg/PLOSOne_formatting_sample_main_body.pdf> and <https://journals.plos.org/plosone/s/file?id=ba62/PLOSOne_formatting_sample_title_authors_affiliations.pdf>

**Response:** We have checked the PLOS ONE’s style requirements and have used the templates to format the manuscript’s title page and main body.

2. Please improve statistical reporting and refer to p-values as "p<.001" instead of "p=.000". Our statistical reporting guidelines are available at <https://journals.plos.org/plosone/s/submission-guidelines#loc-statistical-reporting>.

**Response:** As per your recommendation, we have improved the statistical reporting and referred to *p* values as “p < .001”.

3. Please provide additional details regarding participant consent. In the ethics statement in the Methods and online submission information, please ensure that you have specified what type you obtained (for instance, written or verbal, and if verbal, how it was documented and witnessed). If your study included minors, state whether you obtained consent from parents or guardians. If the need for consent or parental consent was waived by the ethics committee, please include this information.

**Response:** We thank you for your meticulous review. We have included the details regarding the consent form and informed parental consent under the subsections on “Participants” and “Procedures.” Please find the details below.

After:

**Participants (**Page 6, Line 126-Page 7 Line 129**)**

We recruited participants on a nationwide scale from multiple elementary and middle schools in South Korea. Participants were either introduced to this study during their classes or were provided with the information on the bulletin board at school.

**Procedures (**Page 10, Line 211-223**)**

All the participants voluntarily participated in the study. Participants and their parents were informed about the purpose of the study and its procedures. The research assistants provided additional information about the study on the request of the participants or their parents. Written informed consent was obtained from the participants and their parents prior to participation. A URL of the online survey webpage was sent to the participants’ smartphones. Participants logged into a secure webpage and completed a set of self-report measures containing the preliminary versions of the SHSI, K-SHI, CDI, RCMAS, and RSES. It took them approximately 20 minutes to complete all the questionnaires, and they received a monetary reward after submitting the measures. To estimate the temporal stability of the preliminary SHSI, the same tool was administered to 97 participants, four weeks after the first administration. Participants received a gift card worth 5,000 won (approximately 5 dollars) for their participation in the first administration and an additional 3,000 won (approximately 3 dollars) worth gift card after the second administration.

4. Please include your full ethics statement in the ‘Methods’ section of your manuscript file. In your statement, please include the full name of the IRB or ethics committee who approved or waived your study, as well as whether or not you obtained informed written or verbal consent. If consent was waived for your study, please include this information in your statement as well.

**Response:** We have added the full statement in the Method section, including the full name of the IRB and have provided information on the IRB approval, as stated below.

Before

All research procedures were approved by the Institutional Review Board of the authors’ university (IRB NO. KWNUIRB-2019-05-007-002).

**After (Page 10, Line 223-Page 11, Line 225)**

All research procedures were approved by the Institutional Review Board of the Kangwon National University in Chuncheon, South Korea (IRB NO. KWNUIRB-2019-05-007-002).

5. We note that you have referenced (Walsh B, Frost A Unpublished study. 2005.which has currently not yet been accepted for publication. Please remove this from your References and amend this to state in the body of your manuscript: (Walsh B, Frost A Unpublished study. 2005Unpublished study. [2005.as](http://2005.as/) detailed online in our guide for authors <http://journals.plos.org/plosone/s/submission-guidelines#loc-reference-style>

Response: We appreciate your recommendation. We have removed the reference and updated the body of our manuscript as well.

Discussion

Before

Considering the purpose of a screening measure, the SHSI should reduce false-positive errors in selecting adolescents with self-harming behaviors. The excluded four items are not rare but less lethal and, interestingly, were not as popular as more serious forms of self-harm (such as cutting with a knife, carving, hitting head, and burning) in a clinical adolescent sample. (52) These results may be related to the fact that our sample was recruited from a community population in early adolescence.

**After (Page 26, Line 475-478)**

Considering that the previous findings suggest that serious self-harm methods are used among adolescents in clinical setting [55], the SHSI—which considered factor loading as the criteria and not the frequency—might have decreased the false-positive errors in selecting the adolescents with self-harming behaviors.

Reviewer #1: I would like to express my sincere gratitude for the opportunity given to review this manuscript on the development and the validation of the Self-Harm Screening Inventory.
The study’s aim is clearly defined in terms of developing a tool that can help assess or screen non-suicidal self-injury (NSSI) among adolescents in South Korea. The study’s originality is unquestionable, as it varies from other scales or tools developed to screen for NSSI. In addition, the study demonstrates adequate knowledge and understanding of relevant studies on NSSI. However, there are a few aspects of the manuscript that need clarifications.

1. In statements numbered 124 and 125 (pp 6), the authors state that existing measures developed in Western countries have not been validated in Asian countries. This seems to contradict the adaptation study by Kim et al. (2019) (statements 156 &157, pp7).

**Response:** Thank you for your comment. We meant that there is a lack of validated measures that include culturally sensitive items for self-harm in Asia. To clarify the meaning of the text, we have included additional information on self-harm measures in Asian countries as depicted below.

Before (Page 5, Line 123-Page 6 Line 125)

Finally, existing measures were developed in Western countries, and culturally sensitive items have not been developed or validated in Asian countries.

**After (Page 6, Line 107 )**

Finally, the existing measures have primarily been developed in the Western countries, and culturally sensitive items have been seldom developed or validated for use in the Asian countries. In Korea, researchers have translated and validated several measures of self-harm [28-30]. However, no Korean measure has been developed to assess the self-harm behaviors in the Korean population. Likewise, the Japanese version of the DSHI had been validated [31]. However, originally developed Japanese measures to assess self-harm behaviors in Japan, are hard to seek. Although Chinese research has reported a few self-harm measures developed in China, the validation of the measures is rare [32-34]. Considering the potential gap between self-harm behaviors in the Asian and Western cultures, and the lack of reliable tools for assessing specific self-harm behaviors in Asia, it seems necessary to develop items reflecting self-harm behaviors in the Eastern countries.

2. I stand to be corrected but to the best of my knowledge, 1st grade to 3rd grade students are classified as elementary school pupils, not middle school (statements 136-138, pp 6)

**Response:** In South Korea, the grade system is different from that of Western countries. It consists of 1^st^–6^th^ grade in elementary school, 1^st^–3^rd^ grade in middle school, and 1^st^–3^rd^ grade in high school. Therefore, the 1^st^–3^rd^ grade in middle school in South Korea is comparable to 7^th^–9^th^ grade in Western countries. We have added the information in the manuscript to improve readers’ understanding as below.

**After (Page 7, Line 132-133)**

Grade 1-3 in middle school in South Korea is comparable to the grade 7-9 in middle school in Western countries.

3. In the procedure section, little information is given as to how students got to know about the study. Please provide further details on that. In addition, the authors state that monetary reward was given to the participants. Can the authors be specific as to how much was given to each participant to be certain that participants were not enticed to partake in the study but did volunteer.

Response: We thank you for your invaluable advice and have improved on the Method section by incorporating the suggested changes. In the Procedures section, we have added details on advertisement and recruitment, as mentioned below. We have also included the specific amounts for the monetary rewards received by the participants

**Participants**

Before

We recruited participants on a nationwide scale in South Korea.

**After (Page 6, Line 127-Page 7, Line 129)**

We recruited participants on a nationwide scale from multiple elementary and middle schools in South Korea. Participants were either introduced to this study during their classes or were provided with the information on the bulletin board at school.

Procedures

**After (Page 10, Line 211-223)**

All the participants voluntarily participated in the study. Participants and their parents were informed about the purpose of the study and its procedures. The research assistants provided additional information about the study on the request of the participants or their parents. Written informed consent was obtained from the participants and their parents prior to participation. A URL of the online survey webpage was sent to the participants’ smartphones. Participants logged into a secure webpage and completed a set of self-report measures containing the preliminary versions of the SHSI, K-SHI, CDI, RCMAS, and RSES. It took them approximately 20 minutes to complete all the questionnaires, and they received a monetary reward after submitting the measures. To estimate the temporal stability of the preliminary SHSI, the same tool was administered to 97 participants, four weeks after the first administration. Participants received a gift card worth 5,000 won (approximately 5 dollars) for their participation in the first administration and an additional 3,000 won (approximately 3 dollars) worth gift card after the second administration.

4. At the reference section, there are a few “invalid citations” stated. Please review the section and provide the appropriate reference.

Response: We thank you for your comment. We have checked the invalid citations and accordingly modified the manuscript as well.

Nonetheless, sentences are clearly expressed and readable.
Again, I appreciate the opportunity given to review this manuscript and hope to see it in print.

Reviewer #2: The autor does an adequate job elucidating the methodology procedures and data analysis in the manuscript. Overall, the paper is scientifically sound and articulates the content well. However, there are a few questions / elements that need to be addressed. Moreover, there are some mayor edits that are suggested to enhance the text. Comments are provided below, in paragraphs.

1. The title is quite in line with the objective of the research paper; in this regard, perhaps I would highlight the population since the validation wants to be sensitive to it.

**Response:** We thank you for your suggestion. We have included “for adolescents” in the title, to highlight the population under study.

Before

Development and validation of the Self-Harm Screening Inventory (SHSI)

**After**

**Development and validation of the Self-Harm Screening Inventory (SHSI) for Adolescents**

2. The abstract, lacks the hook and the literature gap that you are aiming to contribute to through your paper. For example, it does not highlight the validation goal. It is argued that there is dearth of psychological measures for non-suicidal self-injury (NSSI) screening, but recent literature indicates that there are many and most are for adolescents. In this respect I am not sure that SHI is the most widely used instrument, it would need referencing. I suggest reviewing the current NSSI instrument reviews. Recommend re-visiting it.

Response: We thank you for your meticulous review. We have highlighted the validation goal in the abstract, as mentioned below. Furthermore, we have reworded the text highlighting the lack of culturally appropriate tools for assessing NSSI among Asian adolescents. We also revisited recent literature on self-harm measures and deleted the phrase ‘the most’ (for the use of SHI) from the manuscript.

**Abstract**

Before

Despite the rapidly increasing rate of non-suicidal self-injury (NSSI) among adolescents, there is a dearth of psychological measures for NSSI screening. This study aimed to develop the Self-Harm Screening Inventory (SHSI), a scale for screening NSSI among adolescents.

**After (Page 2, Line 7-Line 9)**

Despite the rapidly increasing rate of non-suicidal self-injury (NSSI) among adolescents, there is a dearth of culturally appropriate psychological measures screening for NSSI among the adolescents in the Asian countries. This study aimed to develop and validate the Self-Harm Screening Inventory (SHSI), a culturally sensitive and suitable scale for screening adolescents for NSSI.

**Page 5, line.**

Before

Among the validated self-report measures, the Self-Harm Inventory (23) is the first and the most widely used measure for assessing self-harming behaviors.

**After (Page 5, Line 83-34)**

One of the valid self-report measures is the Self-Harm Inventory (SHI) [23]; it is the first and widely used measure for assessing self-harm behaviors.

3. NSSI has been clearly defined, however it refers to self-injury as NSSI, self-harm, and other terms. The literature has differentiated NSSI from deliberate self-harm and suicide. If the objective is to screen for NSSI, the instrument should be sensitive to non-suicidal self-injury, but it does not show to be so. I suggest clarifying the terminology and how the instrument is suitable for NSSI screening.

**Response:** We appreciate your thoughtful comment. As the SHSI was developed for screening NSSI, which refers to self-injury without intent to die, instructions were clearly given to the participants. We have added the instructions in the Method section, as stated below.

Before

The Self-Harm Screening Inventory (SHSI) is a brief self-report measure for assessing self-harm behaviors among adolescents. The SHSI consists of 10 binary items (yes/no) which ask about self-harm behaviors within the past year. Cronbach’s alpha of the SHSI in this sample was .795.

After (Page 7, Line 138-147)

**The Self-Harm Screening Inventory (SHSI)**

The Self-Harm Screening Inventory (SHSI) is a brief self-report measure for assessing self-harm behaviors among adolescents. The SHSI consists of 10 binary items (yes/no) which inquire about one’s engagement in self-harm behaviors within the past year. For the purpose of assessing NSSI among adolescents, the definition of NSSI was provided at the end of the instructions as, “Non-suicidal self-injury refers to deliberate self-harm behavior without the intention to die.” The participants were instructed to choose “yes” if they had engaged in self-harm at least once in their lifetime, and to choose “no” if they had not. The Cronbach’s alpha of the SHSI for this sample was .795. The final version of the SHSI is presented in Supplementary Material S1.

4. Overall, the introduction flows well and provides an overview on the remarkable literature. However, I would consider revisiting the introduction and provide a stronger principled argument explaining how your research question will be able to address the gap. Especially regarding the existence of culturally sensitive items and instruments have not been developed or validated in Asian countries.

**Response:** We thank you for your suggestion, and have reviewed sufficient literature to enhance the contents related to the existing culturally sensitive items and instruments, in the Asian countries. Please find the changes below.

Before

Finally, existing measures were developed in western countries, and culturally sensitive items have been developed or validated in Asian countries.

**After (page 6, Line 107-118 )**

Finally, the existing measures have primarily been developed in the Western countries, and culturally sensitive items have been seldom developed or validated for use in the Asian countries. In Korea, researchers have translated and validated several measures of self-harm [28-30]. However, no Korean measure has been developed to assess the self-harm behaviors in the Korean population. Likewise, the Japanese version of the DSHI had been validated [31]. However, originally developed Japanese measures to assess self-harm behaviors in Japan, are hard to seek. Although Chinese research has reported a few self-harm measures developed in China, the validation of the measures is rare [32-34]. Considering the potential gap between self-harm behaviors in the Asian and Western cultures, and the lack of reliable tools for assessing specific self-harm behaviors in Asia, it seems necessary to develop items reflecting self-harm behaviors in the Eastern countries.

5. Methods are generally clearly described and the instruments used seem mostly suitable for their purpose. The statistical approach is adjusted to the development and validation of psychological instruments. However, items have been eliminated in most of the measures for this study. Different measures of NSSI and others have been correlated. If items are removed from an already validated instrument, the data will not be able to be compared between this study and others. Have they been removed because the instrument was not validated for the age group or population of this study? About validation, it would help to justify why it has been validated with the variables with which it has been validated.

**Response:** We thank you for your thoughtful comment. Please find below, the response for both your questions.

1) Have they been removed because the instrument was not validated for the age group or population of this study?

**Response:** No, this study aimed to develop a brief screening measure for self-harming behaviors among adolescents. Thus, we selected the most relevant and informative items among the original items to screen the participants for NSSI.

2)  About validation, it would help to justify why it has been validated with the variables with which it has been validated.

-----Messick (1989) recommended five criteria for validation, as under content, construct, responses, criterion, and consequence evidence. To validate the scale, the criterion evidence was used. It is "validated with the variables with which it has been validated"

If the developed scale is correlated with the scale which it has been validated, the developed scale can be validated.

Reference

Messick, S. (1989). Validity. In R. Linn (Ed.). Educational Measurement (3rd edition) (pp.13-100). Washington, DC: American Council on Education.

6. The discussion is according to the objectives and results. The selection criteria based on expert has clearly delimited the items that are more serious, this could make the SHSI measure less prevalence of NSSI than many of the commonly used instruments. It would be necessary to demonstrate through literature the need to agree on this criterion. On the other hand, it also seems necessary to make explicit in the text that the instrument measures the most serious methods. Furthermore, it should be discussed and also included in a more extended discussion of how SHSI improves NSSI screening.

Response: We thank you for your thoughtful comment. For the development of a quick screening tool, we adopted two methods: qualitative investigation and quantitative investigation. In the qualitative investigation, we asked the experts in the field of self-harm and suicide, to rate the appropriateness, sensitivity and prevalence of each of the NSSI behaviors. Their ratings were not based on the severity or lethality of the behavior. After selecting 20 items based on the expert’s ratings, we used exploratory factor analysis to determine the final 10 items. In the quantitative investigation, we used factor loadings and not the frequency of each item, as the selecting criteria. To clarify the procedures and the meaning, we have modified the manuscript as well, as stated below.

**Procedures (Page 9, Line 179-**

For the development of the preliminary SHSI, multiple sources of information were reviewed. Previous research findings (academic sources), news, and social media (such as Twitter, Instagram, Facebook, and Tumblr), describing self-harm behaviors and related clinical observations, were investigated. The comments of experts on self-harm and suicide attempts (non-academic sources) were also considered. Further, the items assessing self-harm behaviors in the pre-existing self-harm measures, such as the SHI, DSHI, and FASM, were collected. The focus of the literature review was to generate a list of specific and observable NSSI behaviors. Thereafter, the overlapping items and dimensions in the list were either combined or reorganized.

A total of 28 items were developed for the first version of the preliminary measure (Supplemental Material S2) and sent to 11 Korean experts—four clinical psychologists, five psychiatrists, and two school counselors —in the areas of self-harm and suicide. The experts rated individual items for their *appropriateness* (in assessing self-harm among adolescents), *sensitivity* (to distinguish the adolescents engaging in serious self-harm), and *prevalence* (of the behavior among Korean adolescents). They rated each item on a seven-point Likert scale (1 = very unlikely; 7 = very likely) for *appropriateness* and *sensitivity*, and a three-point Likert scale (1 = low prevalence; 3 = high prevalence) for *prevalence*. They also provided additional comments on the utility of the items. Thereafter, the mean rating scores for each item were calculated. First, items with scores lower than 5 for *appropriateness* and *sensitivity*, and scores lower than 2 for *prevalence*, were identified. Of the 28 items, eight items below the aforementioned scores were identified under all three dimensions, one item under two of the dimensions, and six items under only one dimension. Items identified in more than two of the dimensions were excluded. Second, the informative value of the excluded items was reconsidered in the ensuing discussion among the authors. Of the nine items identified under more than two dimensions, the item on “overdose” was considered a distinctive and comparatively common method of self-harm. It was, thus, re-included among the preliminary items. Consequently, eight items with low scores on more than two of the dimensions (appropriateness, sensitivity, and prevalence) were discarded before administration (e.g., items on Drank excessively, Engaged in unhealthy sexual relationships (e.g., having multiple partners), Had unsafe sex, Starved to the point of harming your body (for a few days at least), Took laxatives, diuretics, etc. to the point of harming your body, Rubbed skin on rough surfaces, Swallowed dangerous substances (bleach, hand sanitizer, detergent, etc.), Got a tattoo). Twenty items with higher scores and utility were retained as preliminary items for the SHSI and administered to the participants along with other self-report measures.

Results (Page 16, Line 305-309)

The final structure showed a good model fit: *χ²* (119) = 207.189, *p* < .001, RMSEA = .038, CFI = .964, TLI = .959, WRMR = 1.154. Factor loadings for the total items ranged between .555 and .985. Among the 17 items, 10 items (preliminary items 1, 2, 3, 4, 6, 7, 15, 17, 19, and 20), which showed high factor loadings (greater than 0.7), were examined by two authors for content validity, and were selected for the final version of the SHSI.

**Discussion**

Before (Page 27, Line )

Considering the purpose of a screening measure, the SHSI should reduce false-positive errors in selecting adolescents with self-harming behaviors. The excluded four items are not rare but less lethal and, interestingly, were not as popular as more serious forms of self-harm (such as cutting with a knife, carving, hitting head, and burning) in a clinical adolescent sample. (52) These results may be related to the fact that our sample was recruited from a community population in early adolescence.

**After (Page 26, Line 475-478)**

Considering that the previous findings suggest that serious self-harm methods are used among adolescents in clinical setting [55], the SHSI—which considered factor loading as the criteria and not the frequency—might have decreased the false-positive errors in selecting the adolescents with self-harming behaviors.

7. Finally, a few minor comments:

Line 285: “scree plot”
Response: We are unsure as to what you are suggesting for the “scree plot”. Please let us know what your recommendation is.

Line 314 and 320-322: The data is already in the table, perhaps it would help the reader if it were evaluated instead of repeating the data.

**Response:** We thank you for your suggestion and have modified the manuscript accordingly.

Line 314

Before

The item-total correlations for each item ranged between .310 and .670, and Cronbach’s alpha was .795.

**After (Page 21, Line 352-353)**

The item-total correlations for each item were acceptable, ranging between .310 and .670. The Cronbach’s alpha was good (α = .795).

Line 320-322

Before

The SHSI had high correlations with the SHI (*r* = .775, *p* < .01), and showed moderate correlations with the CDI (*r* = .489, *p* < .01) and RCMAS (*r* = .433, *p* < .01). The SHSI negatively correlated with the RSES (*r* = -.399, *p* < .01; Table 7).

**After (Page 21, Line 359-363)**

The SHSI had high correlations with the SHI (*r* = .775, *p* < .01), and moderate correlations with the CDI (*r* = .489, *p* < .01) and RCMAS (*r* = .433, *p* < .01). The SHSI was negatively correlated with the RSES (*r* = -.399, *p* < .01; Table 7). The results indicate that SHSI is a reliable and valid measure for NSSI, and it is distinct from the other psychological measures assessing depression, anxiety, and self-esteem.

Line 342: The FASM items selection process was described in in the author's doctoral thesis.

**Response:** We thank you for your comment. We have looked up the doctoral thesis of the author (Lloyd, 1998) and found that the author “established a list of self-mutilative behaviors based upon review of previous research (Ross & McKay, 1979; Walsh & Rosen, 1988), as well as the authors’ clinical experience.” However, detailed information on the process of selecting the items was not presented in the doctoral thesis either.

Reviewer #3: This is very interesting paper regarding NSSI issues and wih this new itmes, clinicinas can assess NSSI patients more systematically and effectively. However, I have several concerns.

1. Please check reference 8 which is inapporpiriate.

**Response:** We had cited the estimates of prevalence among clinical inpatient adolescents in the reference 8 (Hamza et al., 2008). The cited part was as below.

*“Estimates of prevalence suggest that among clinical inpatient samples, as many as 21% of adults (**[Briere & Gil, 1998](https://www.sciencedirect.com/science/article/pii/S0272735812000669?casa_token=VNL27Cf5cNMAAAAA:ecHtJGiK-WcL82n8YWfN0m2U-5GUcqWT6L3SXfb6IBKCpXQZgrdwM03sAiUIuxJEYlMBM8qpPuut#bb0055)) and 30 to 40% of adolescents engage in NSSI (**[Darche, 1990](https://www.sciencedirect.com/science/article/pii/S0272735812000669?casa_token=VNL27Cf5cNMAAAAA:ecHtJGiK-WcL82n8YWfN0m2U-5GUcqWT6L3SXfb6IBKCpXQZgrdwM03sAiUIuxJEYlMBM8qpPuut#bb0105),* *[Jacobson et al., 2008](https://www.sciencedirect.com/science/article/pii/S0272735812000669?casa_token=VNL27Cf5cNMAAAAA:ecHtJGiK-WcL82n8YWfN0m2U-5GUcqWT6L3SXfb6IBKCpXQZgrdwM03sAiUIuxJEYlMBM8qpPuut#bb0220)). NSSI is not only a clinical health concern, however, as recent estimates based on community samples indicate that as many as 13 to 29% of adolescents (**[Baetens et al., 2011](https://www.sciencedirect.com/science/article/pii/S0272735812000669?casa_token=VNL27Cf5cNMAAAAA:ecHtJGiK-WcL82n8YWfN0m2U-5GUcqWT6L3SXfb6IBKCpXQZgrdwM03sAiUIuxJEYlMBM8qpPuut#bb0030),* *[Brausch and Gutierrez, 2010](https://www.sciencedirect.com/science/article/pii/S0272735812000669?casa_token=VNL27Cf5cNMAAAAA:ecHtJGiK-WcL82n8YWfN0m2U-5GUcqWT6L3SXfb6IBKCpXQZgrdwM03sAiUIuxJEYlMBM8qpPuut#bb0050),* *[Heath et al., 2007](https://www.sciencedirect.com/science/article/pii/S0272735812000669?casa_token=VNL27Cf5cNMAAAAA:ecHtJGiK-WcL82n8YWfN0m2U-5GUcqWT6L3SXfb6IBKCpXQZgrdwM03sAiUIuxJEYlMBM8qpPuut#bb0180),* *[Ross and Heath, 2002](https://www.sciencedirect.com/science/article/pii/S0272735812000669?casa_token=VNL27Cf5cNMAAAAA:ecHtJGiK-WcL82n8YWfN0m2U-5GUcqWT6L3SXfb6IBKCpXQZgrdwM03sAiUIuxJEYlMBM8qpPuut#bb0400)) and 4–6% adults engage in NSSI (*[*Briere and Gil, 1998*](https://www.sciencedirect.com/science/article/pii/S0272735812000669?casa_token=VNL27Cf5cNMAAAAA:ecHtJGiK-WcL82n8YWfN0m2U-5GUcqWT6L3SXfb6IBKCpXQZgrdwM03sAiUIuxJEYlMBM8qpPuut#bb0055)*,* *[Klonsky, 2011](https://www.sciencedirect.com/science/article/pii/S0272735812000669?casa_token=VNL27Cf5cNMAAAAA:ecHtJGiK-WcL82n8YWfN0m2U-5GUcqWT6L3SXfb6IBKCpXQZgrdwM03sAiUIuxJEYlMBM8qpPuut#bb0245)).”*

Also, the authors should correct or replace the references regarding korean newspapers which is only written in Korean and which is not found with using even internet adress such as ref. 14, 19, 20, and etc. The authors should support their ideas with more evidenced and English-written publications, since this journal is internationally read.

**Response:** We thank you for your thoughtful comment. Since the SHSI was developed as a culturally sensitive measure for the adolescent population, we have not only cited references written in English but also those written in Korean. Due to the limited academic evidences on the novel phenomena related to NSSI (e.g., engaged in bloodletting) among adolescents in Korea, especially those different from Western findings, we have included Korean newspapers. We have checked the online address of the articles once again and modified the reference as below.

Before

16.	Lee JH, Choe, Y. Y., Jeon, J. Y. . "self-harm experience" in 70,000 middle school and high school students 2018. November, 11th. [Available from: <http://www.hani.co.kr/arti/society/> society_general/869668.html.

19.	W. YJ. Adolescents cousulted with self-harm has been tripled. Yonhap News. 2019. 05. 29.

21.	Edujin. Is your 'friend' safe, now? Nachimban. 2018. 11. 09.

22.	Song HS. Warning for upsurge of self-harming adolescents. Shindonga. 2019. April 7th.

After

16. Lee JH, Choe YY, Jeon JY. "Self-harm experience" in 70,000 middle school and high school students. The Hankyoreh. 2018 Nov 11 [Cited 2021 Oct 15]. Available from: <https://www.hani.co.kr/arti/society/society_general/869668.html>

19. Yang, JW. Adolescents consulted with self-harm have been tripled. The Yonhap News. 2019 May 29 [Cited 2021 Oct 15]. Available from: <https://www.yna.co.kr/view/AKR20190529078500005>.

21. Moon YH, Is your ‘friend’ safe, now?. The Edujin Nachimban. 2018 Nov 9 [Cited 2021 Oct 15]. Available from: <http://www.edujin.co.kr/news/articleView.html?idxno=20216>

22. Song HS. Warning for upsurge of self-harming adolescents. The Shindonga. 2019 Apr 7 [Cited 2021 Oct 15]. Available from: <https://shindonga.donga.com/3/all/13/1686942/1>.

2. In the introduction, I cannot understand what the authors mean by the difference between self-injury and self-harm in page 3, line 64. Please clarify the meaning or delete the sentence.

**Response:** Thank you for your valuable comment. We have removed “self-harm” and clarified the meaning of the sentence as below.

Before

Girls are known to show NSSI earlier than boys are at higher risk of developing self-harm during adolescence. NSSI declines over time, but approximately 20% of adolescents who start NSSI maintain the behavior for more than five years, and it often develops into a chronic and malignant practice

**After (Page 3, Line 38-42)**

Girls are known to show NSSI earlier than boys and are at a higher risk of developing self-harm behaviors during adolescence [5,9,10]. NSSI declines over the course of its development from adolescence to early adulthood. However, approximately 20% of adolescents maintain the behavior for more than five years, and it often develops into a chronic and malignant practice that lasts until adulthood [4].

3. In the method section, I recommand the authors should show the readers how 28 items were selected from the scales the authors utilized, more clearly. For this purpose, the authors can use the supplemental enlisting materials.

**Response:** We thank you for your thoughtful suggestion. We have added the preliminary 28 items under the Supplemental Material S2.

Before

For the development of the preliminary SHSI, multiple sources of information were reviewed. Previous research findings (academic sources), news and social media contents describing self-harm behaviors, and clinical observations and comments of experts in self-harm and suicidal attempts (non-academic sources) were investigated. Further, items from pre-existing self-harm measures, such as SHI, DSHI, and FASM, were collected. Overlapping items and areas were combined or reorganized. A total of 28 items were developed for the first version of the preliminary measure, and sent to 11 Korean experts in self-harm and suicide: four clinical psychologists, five psychiatrists, and two school counselors.

**After (Page 9, Line 179-192)**

For the development of the preliminary SHSI, multiple sources of information were reviewed. Previous research findings (academic sources), news, and social media (such as Twitter, Instagram, Facebook, and Tumblr), describing self-harm behaviors and related clinical observations, were investigated. The comments of experts on self-harm and suicide attempts (non-academic sources) were also considered. Further, the items assessing self-harm behaviors in the pre-existing self-harm measures, such as the SHI, DSHI, and FASM, were collected. The focus of the literature review was to generate a list of specific and observable NSSI behaviors. Thereafter, the overlapping items and dimensions in the list were either combined or reorganized.

A total of 28 items were developed for the first version of the preliminary measure (Supplemental Material S2) and sent to 11 Korean experts—four clinical psychologists, five psychiatrists, and two school counselors —in the areas of self-harm and suicide. Furthermore, to ease comprehension among the readers, we have added the preliminary 28 items under the Supplemental Materials.

[Supplemental Material_ S2 Table 2]

**S2 Table 2. The Preliminary 28 items of the self-harm behaviors among adolescents**

| No. | Item |
| --- | --- |
| 1 | Overdosed on drugs. |
| 2 | Cut my body with sharp objects. |
| 3 | Hit my body (e.g. hitting my body, such as the head, hard with my hands). |
| 4 | Banged my head against a wall, desk, etc. |
| 5 | Hit things hard with my fist. |
| 6 | Drank excessively. |
| 7 | Scratched my body. |
| 8 | Engaged in unhealthy sexual relationships (e.g., having multiple partners). |
| 9 | Had unsafe sex. |
| 10 | Starved to the point of harming my body (for a few days at least). |
| 11 | Took laxatives, diuretics, etc. to the point of harming my body. |
| 12 | Cut or carved something onto skin using a knife. |
| 13 | Pulled my hair out. |
| 14 | Picked or pinched my wound. |
| 15 | Burnt my skin with fire (using a cigarette, match, or other hot objects). |
| 16 | Stuck objects underneath my fingernails or into my skin. |
| 17 | Bit parts of my body (e.g., mouth and lips) |
| 18 | Scratched my skin until it left scars. |
| 19 | Picked or peeled off my skin. |
| 20 | Stabbed my body with sharp or pointed objects. |
| 21 | Cut holes in my body (e.g. not ear piercings or body piercings that are meant to enhance beauty, but to make holes to harm myself) |
| 22 | Slit or cut my body with sharp objects |
| 23 | Carved words or symbols onto my body. |
| 24 | Rubbed my skin on rough surfaces. |
| 25 | Swallowed dangerous substances (bleach, hand sanitizer, detergent, etc.). |
| 26 | Strangled my neck. |
| 27 | Engaged in bloodletting (drained blood from my body). |
| 28 | Got a tattoo. |

4. In the method section, the authors describled the exclusion and inclusion processes they made. However, it is hard to read and understand full process clerarly. With using addtional figure, the readers are able to understand your process more clearly.

Response: We thank you for your recommendation, and have added a figure which depicts the research flow, as shown below.

*Fig 1. Flow of the research*

5. I also wonder what was first 20 items in detail.

Response: We thank you for your question. Please find preliminary 20 items in Table 1 of the manuscript.
